# Case Report: An undescended intravagal parathyroid adenoma: a rare cause of refractory primary hyperparathyroidism and severe osteoporosis

**DOI:** 10.3389/fendo.2026.1783095

**Published:** 2026-02-18

**Authors:** Hassan A. Alzahrani

**Affiliations:** Department of General Surgery, College of Medicine, King Khalid University, Abha, Saudi Arabia

**Keywords:** ectopic, intravagal, parathyroid adenoma, primary hyperparathyroidism, vagus nerve

## Abstract

Ectopic intravagal parathyroid adenomas represent an exceedingly rare clinical entity. Embryologically, parathyroid glands develop from the third and fourth pharyngeal pouches, migrating to their definitive locations on the inferior and superior aspects of the thyroid. While ectopic locations are well described, intravagal variants are rare, with few documented cases in the literature. We report an additional unique case of a patient with persistent hypercalcemia and severe osteoporosis following a failed bilateral neck exploration for primary hyperparathyroidism. Following multimodal structural and functional radiological evaluation, an ectopically undescended parathyroid adenoma was identified and subsequently confirmed intraoperatively to be situated intravagal.

## Introduction

During embryonic development, the parathyroid glands originate from the third and fourth pharyngeal pouches and migrate to their designated positions on the dorsal side of the thyroid, specifically at the inferior and superior sites (1). Any disruption in this migration may result in ectopic parathyroid tissue forming along the migratory pathway or within adjacent cervical structures. Ectopic parathyroid tissue is a well-recognized phenomenon, previously reported in various locations, including the carotid sheath and the middle mediastinum (2). Awareness of these aberrant locations is clinically imperative, as parathyroid adenomas typically necessitate precise surgical intervention. While ectopic parathyroid adenomas are extensively described, intravagal parathyroid adenomas remain exceptionally rare, with only few cases documented in the literature (3). In this report, we describe the identification, surgical management, and postoperative outcomes of a unique case involving a parathyroid adenoma located within the vagus nerve.

## Case presentation

A 39-year-old female was referred to the endocrine surgery clinic for the evaluation of persistent primary hyperparathyroidism. One year prior, she had undergone an unsuccessful bilateral neck exploration at another facility. Upon presentation, her serum calcium was 11.3 mg/dL, and she reported debilitating fatigue and bone pain. Physical examination revealed that the patient was chair-bound due to multiple pathological fractures, with a well-healed surgical scar on the neck.

**Figure 1:**
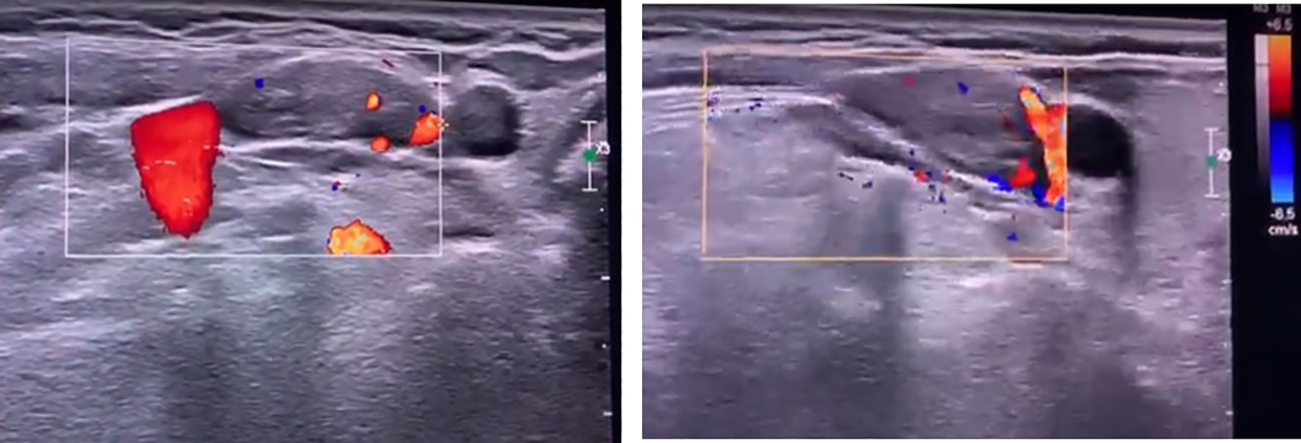
Ultrasound images showing a hypoechoic mass, medial to the internal jugular vein and posteriolateral to the carotid artery.

**Figure 2:**
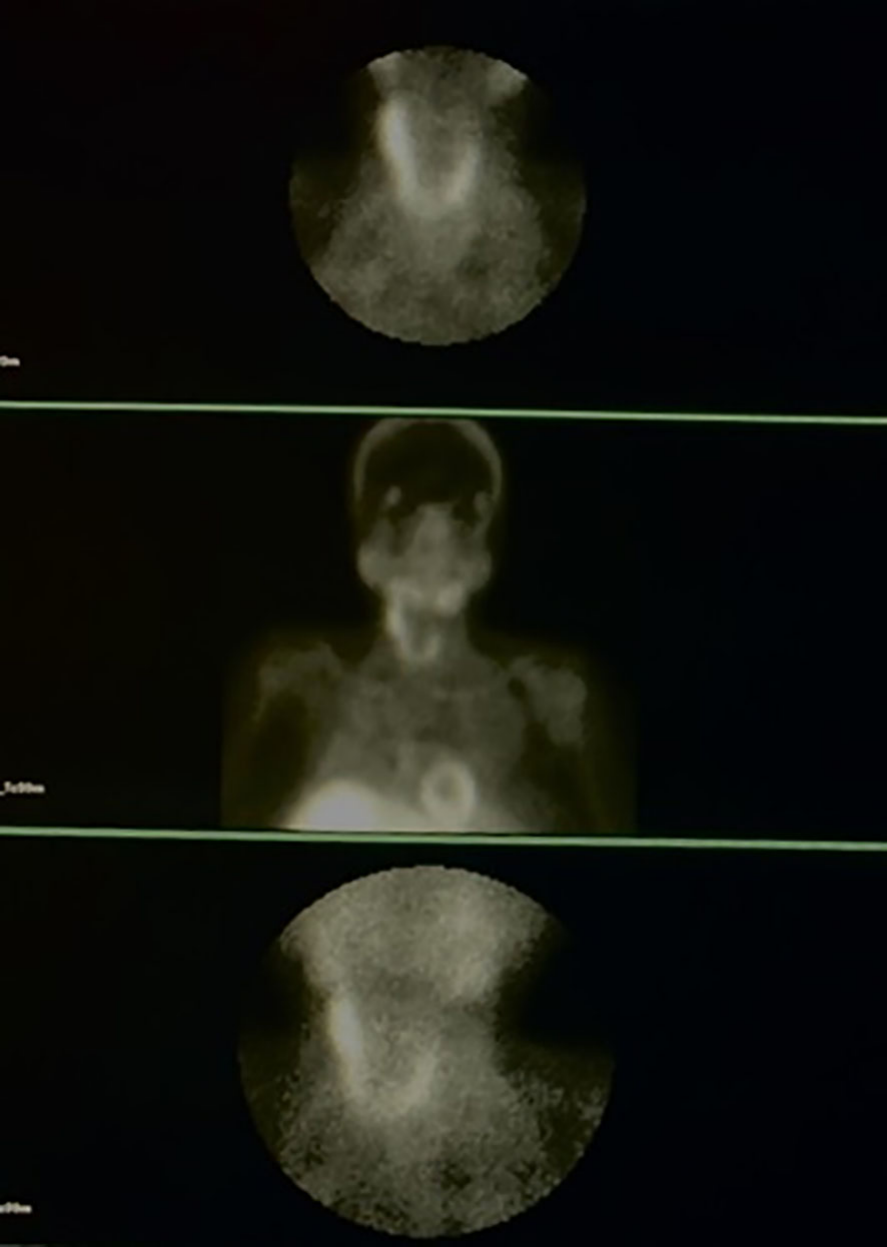
Sestamibi scan images illustrating an uptake in right undescended parathyroid tissue.

**Figure 3:**
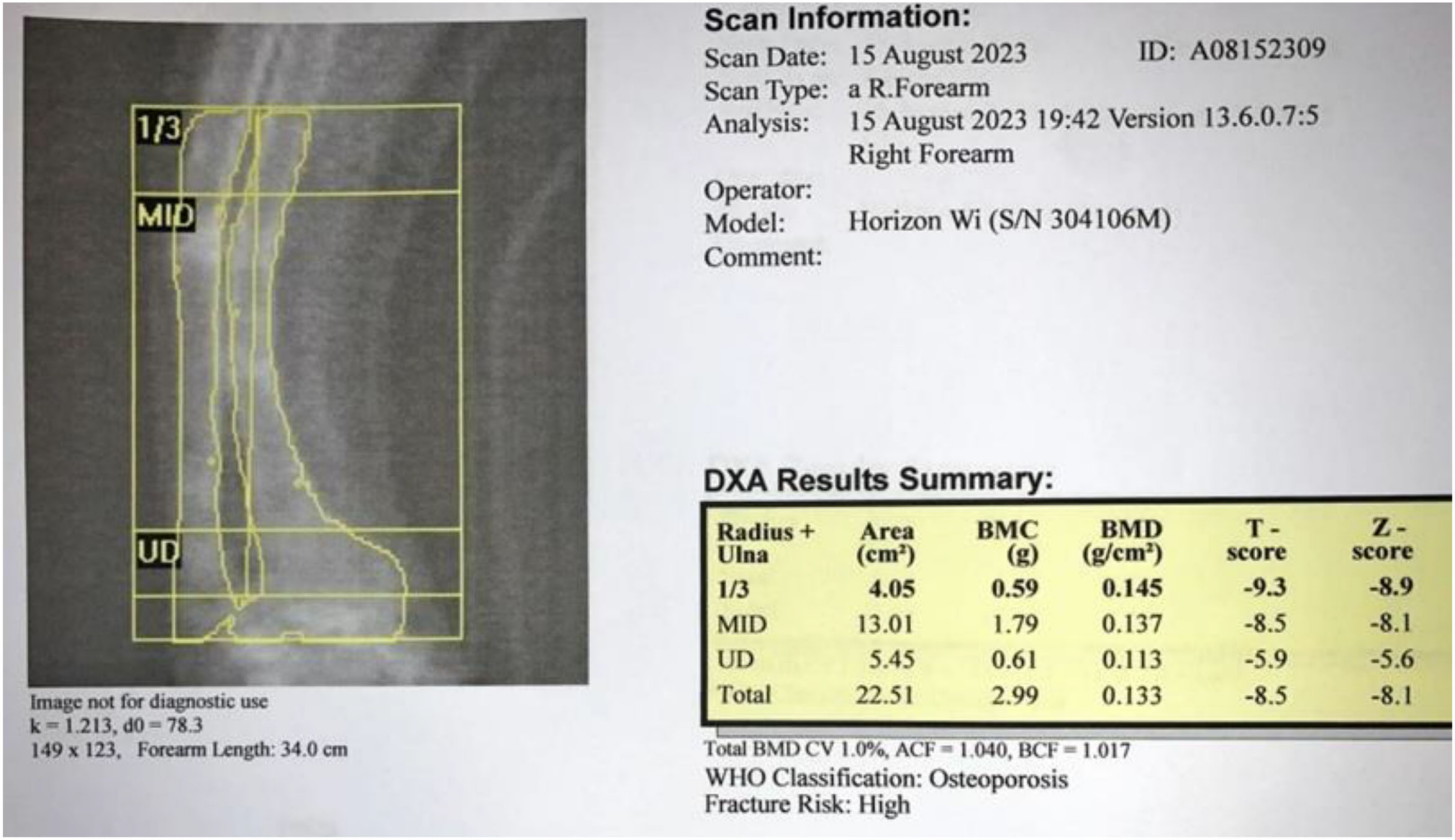
Forearm DEXA scan image/report prior to surgery showing very severe osteoporosis.

**Figure 4:**
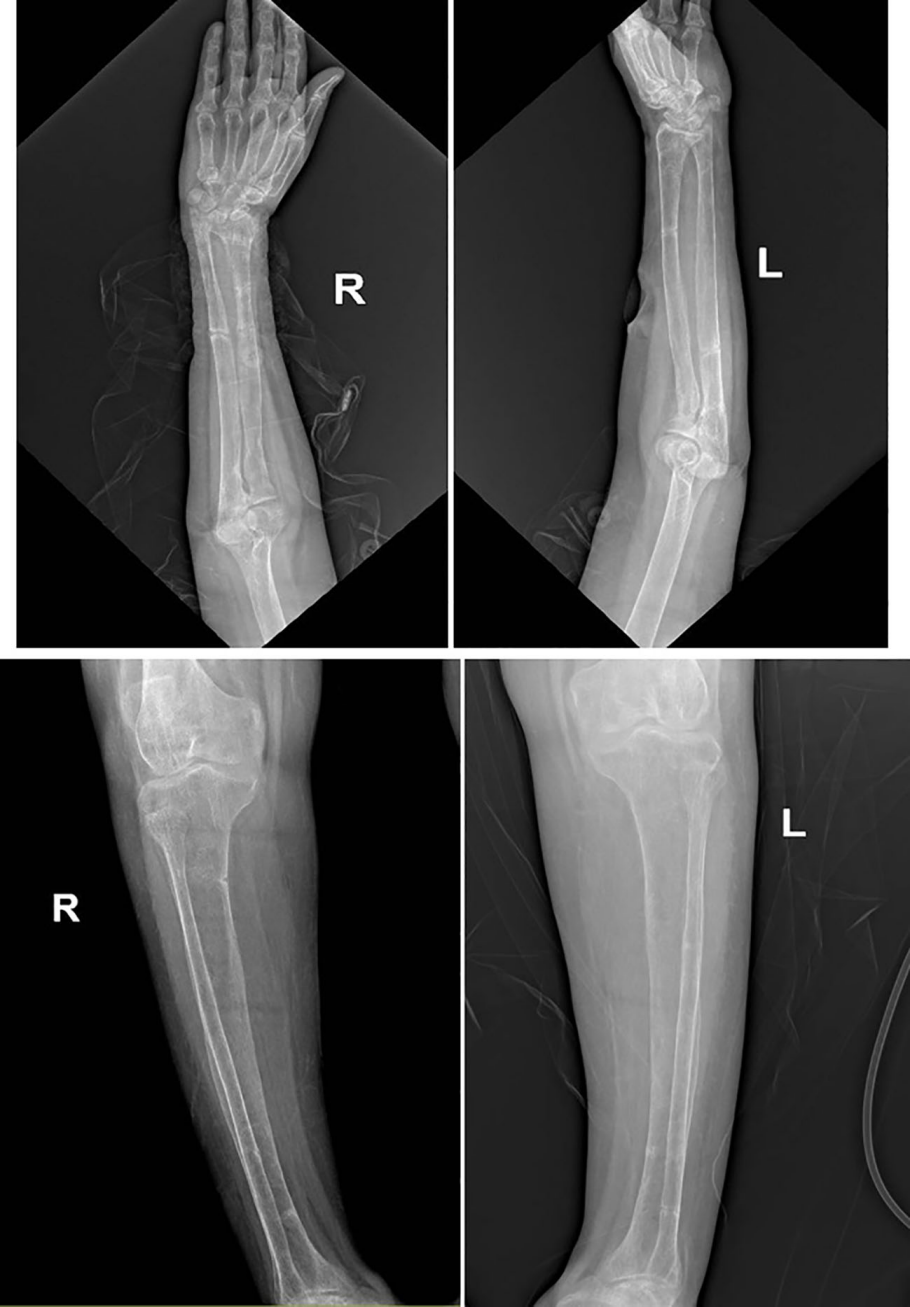
Skeletal radiographic images showing multiple fractures involving upper and lower limbs.

Comprehensive neck ultrasonography identified a hypoechoic mass at the level of the right carotid bifurcation, situated medial to the internal jugular vein and posteriolateral to the carotid artery (**Figure 1**). A functional parathyroid scan utilizing intravenous Technetium-99m (99mTc) sestamibi demonstrated focal tracer uptake within the right carotid sheath, correlating with the sonographic findings (**Figure 2**). Furthermore, a dual-energy X-ray absorptiometry (DEXA) scan of the distal one-third of the forearm revealed profound osteoporosis with a T-score of -9.3 (**Figure 3**). Corresponding skeletal radiographs confirmed generalized osteopenia and several fragility fractures (**Figure 4**).

**Figure 5:**
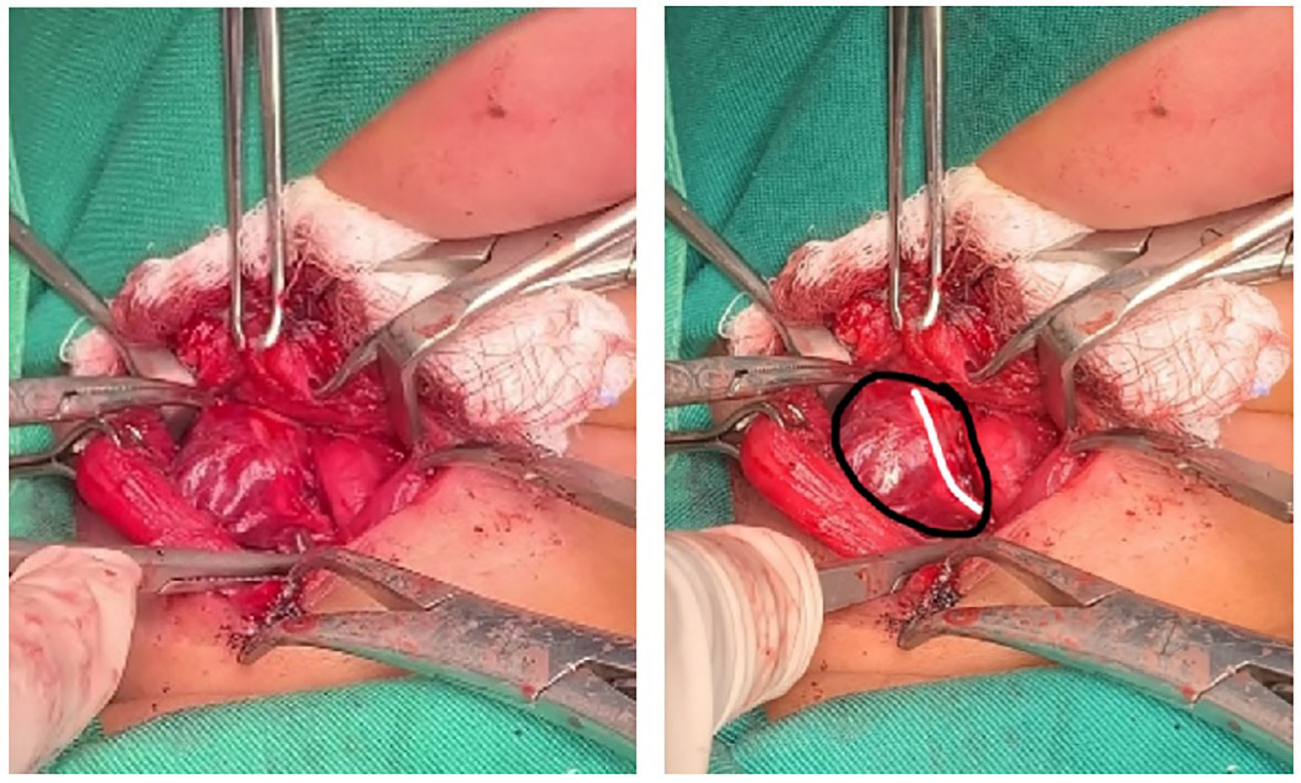
An intraoperative picture showing intravagal parathyroid adenoma (Black circle illustrate the parathyroid adenoma and white line illustrate the vagal nerve).

**Figure 6:**
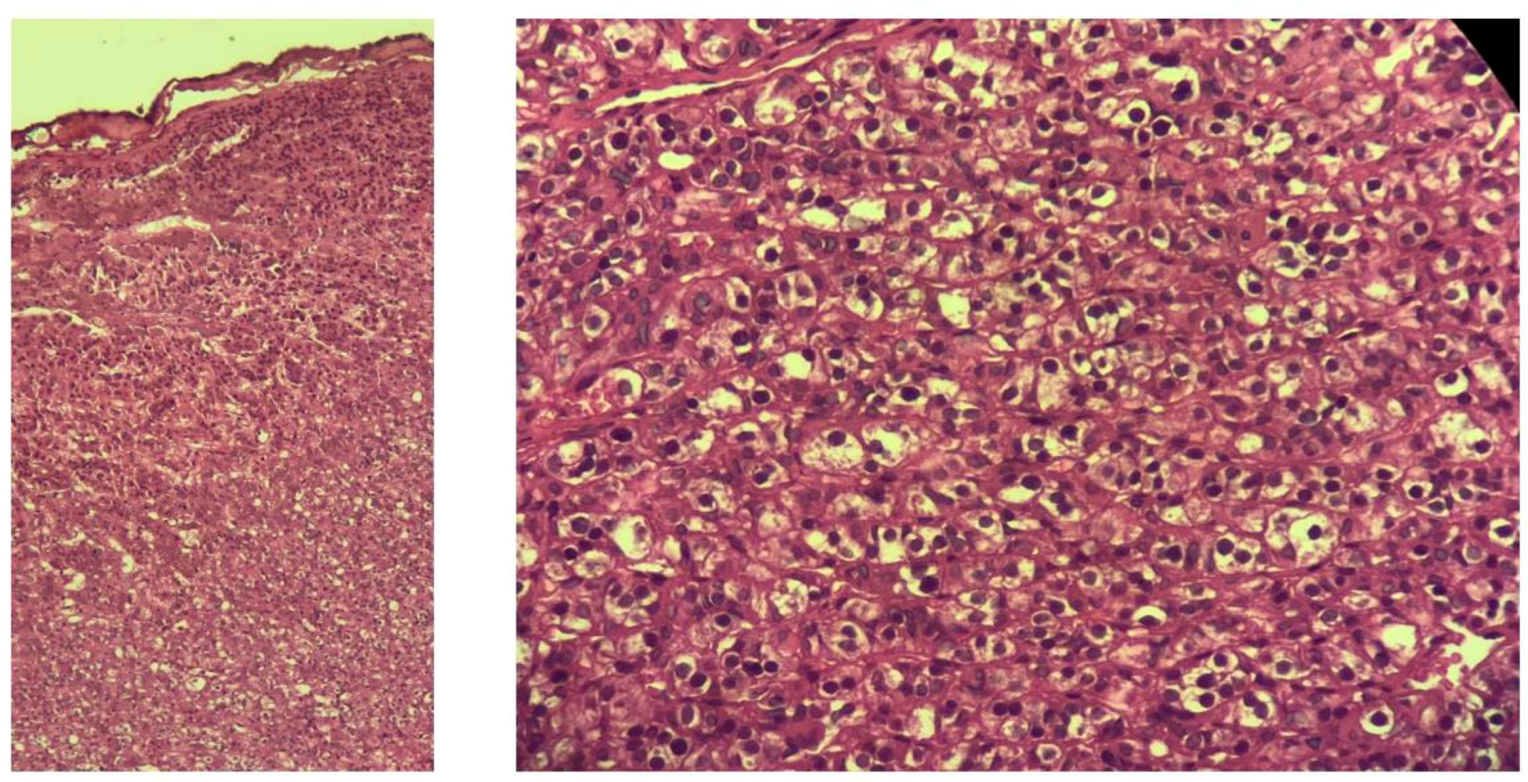
Histopathological images confirming the presence of hypercellular parathyroid tissue (hematoxylineosin, H&E, stain).

A redo parathyroidectomy was performed via a focused transcervical approach. Intraoperatively, a 2.9 x 0.8 x 0.4 cm mass was identified within the right vagus nerve (**Figure 5**). The adenoma was meticulously dissected from the nerve sheath in toto, preserving neural integrity. Following excision, intraoperative parathyroid hormone (IOPTH) levels plummeted from a baseline of 960 pg/mL to 65 pg/mL, a drop of over 90%. Histopathological examination subsequently confirmed the tissue as a parathyroid adenoma (**Figure 6**).

**Figure 7:**
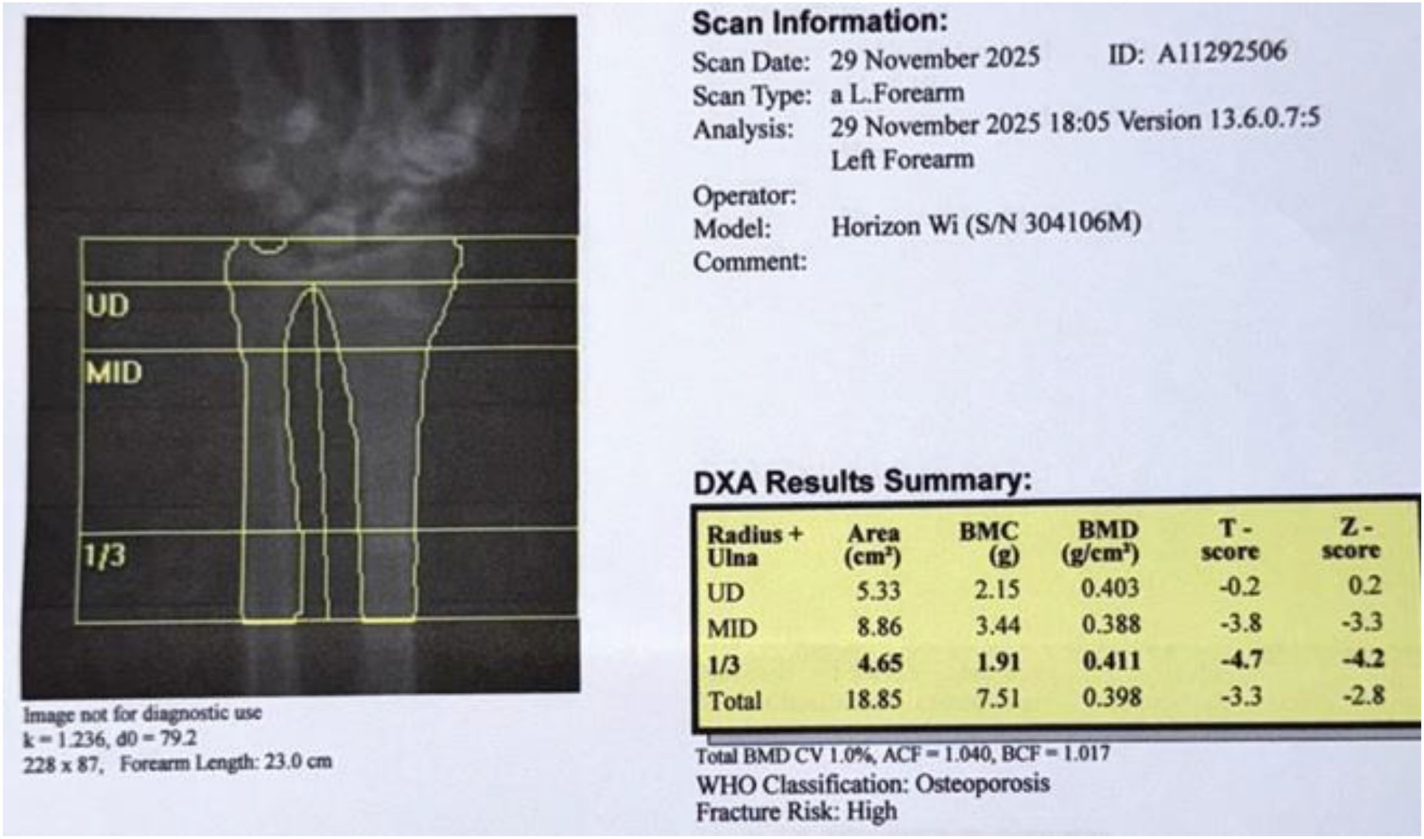
Forearm DEXA scan image/report after 2 years of surgery showing significant improvement.

Due to the severity of the preoperative bone disease, the patient was closely monitored for Hungry Bone Syndrome, which was managed successfully. She was discharged on the fifth postoperative day with stable calcium levels, good oral intake, no aspiration, and a normal voice, supported by a regimen of elemental calcium and vitamin D. At the two-year follow-up, the patient’s clinical status had improved significantly; she achieved assisted ambulation, and her bone mineral density showed dramatic recovery, with the forearm DEXA T-score improving from -9.3 to -4.7 (**Figure 7**).

## Discussion

The parathyroid glands originate from endodermal epithelial cells in close developmental association with the thymus. The superior parathyroid glands arise from the fourth branchial (pharyngeal) pouch and typically maintain a consistent anatomical relationship with the lateral thyroid lobes due to their relatively short migratory path. In contrast, the inferior parathyroid glands derive from the third branchial pouch and migrate alongside the thymus. This significantly longer line of embryologic descent results in a much higher degree of variability in their final anatomical position (2).

Ectopic parathyroid glands arise from the co-migration of parathyroid tissue with embryologically related structures during development. When a gland fails to complete its typical migratory path, it is classified as “undescended”. These ectopic presentations may involve any of the four standard glands or may manifest as supernumerary tissue, which is reported in approximately 13% to 15% of cases. In clinical series of patients requiring reoperation for persistent or recurrent hyperparathyroidism, ectopic glands have been localized to various anatomical compartments: the paraesophageal space (28%), the mediastinum (26%), and the thymus (24%). Less frequent sites include the thyroid parenchyma (11%), the carotid sheath (9%), and high cervical positions (2%) (4).

The embryologic foundation for ectopic parathyroid tissue within the vagus nerve was established by Gilmour in 1937. Through fetal dissections, he identified accessory parathyroid tissue sequestered within the vagal ganglion, attributing this to the close developmental proximity between the third branchial pouch and the vagus nerve. Gilmour hypothesized that parathyroid fragments could detach and become embedded within the nerve during embryonic migration, a theory subsequently corroborated by the findings of Lack et al. in 1988. Clinical evidence provided by Pawlik et al. further aligns with these observations, describing cases where adenomas were situated below the vagal ganglia and several centimeters inferior to the carotid bifurcation (5).

Ectopic parathyroid adenomas should be considered in patients with primary hyperparathyroidism when imaging suggests an ectopic location, initial imaging fails to find an adenoma, or initial surgery is unsuccessful (6). This diagnostic challenge is further intensified in the context of Multiple Endocrine Neoplasia Type 1 (MEN1), where ectopic adenomas and supernumerary glands are distinguishing clinical hallmarks. As a rare genetic disorder, MEN1-associated primary hyperparathyroidism typically manifests with greater clinical severity and an earlier age of onset than sporadic cases. Surgery in these complex cases should be performed by experienced, high-volume surgeons for optimal outcomes, typically involving subtotal parathyroidectomy (removal of 3 to 3.5 glands) to mitigate surgical failure and recurrence risk, while preventing permanent hypoparathyroidism associated with these complex clinical presentations (7).

The occurrence of ectopic intravagal parathyroid adenomas underscores the intricate nature of parathyroid embryology and the critical importance of recognizing anatomical variations in clinical practice. This case expands the sparse literature regarding intravagal parathyroid adenomas (3), highlighting the necessity for clinicians to maintain a high index of suspicion for such rare variants in patients with persistent hyperparathyroidism—particularly those with a history of unsuccessful surgical exploration.

In the present case, the diagnosis was established through a synthesis of clinical presentation, biochemical markers, and targeted imaging. Specifically, the integration of ultrasonography and nuclear medicine scintigraphy was pivotal in localizing the ectopic tissue within the carotid sheath. These findings further substantiate the essential role of multimodal imaging protocols in navigating the diagnostic challenges of persistent primary hyperparathyroidism.

Glasgow et al. summarized several imaging methods for localizing ectopic parathyroid glands, including ultrasound, ultrasound guided fine needle aspiration (FNA), parathyroid scintigraphy, four-dimensional CT (4D-CT), Positron Emission Tomography (PET) using 11C-methionine or 18F-choline (6).

While ultrasound is a common initial step, it demonstrates low sensitivity for glands in mediastinal or paraesophageal regions. Similarly, ultrasound-guided FNA for cytology and parathyroid hormone levels is valuable but must be avoided if parathyroid carcinoma is clinically suspected. Parathyroid scintigraphy, utilizing technetium-99m (99mTc) sestamibi, relies on the tracer’s retention in mitochondria-rich oxyphil cells. However, its efficacy is often hindered by tracer concentration in the thyroid or submandibular glands, or by interference from multinodular goiters and retropharyngeal adenomas. To overcome these limitations, 4D-CT analyzes perfusion characteristics across multiple contrast phases, while PET using 11C-methionine or 18F-choline offers higher sensitivity, though its availability remains limited to specialist centers.

International endocrinology and surgical societies increasingly recommending 18F-choline PET as a superior nuclear imaging test for suspected ectopic cases and MEN1-associated primary hyperparathyroidism (7).

Utilizing a meticulous, focused transcervical approach, the intravagal adenoma was successfully excised, leading to an immediate and significant reduction in IOPTH levels. However, the intimate proximity of these tumors to the vagus nerve and adjacent neurovascular structures introduces substantial surgical risks. Potential postoperative morbidities associated with vagal dissection include recurrent laryngeal nerve palsy or Horner’s syndrome, typically resulting from nerve manipulation or the necessity of vagal sacrifice (3).

The immediate postoperative improvement in hypercalcemia and the patient’s subsequent recovery underscore the effectiveness of surgical re-exploration in cases of ectopic parathyroid adenomas. Moreover, the notable improvement in bone density (8), evidenced by DEXA scan, points to the long-term benefits of addressing underlying hyperparathyroidism, which can lead to better quality of life (9), and functional independence (10).

## Conclusion

This case underscores the significance of recognizing ectopic intravagal parathyroid adenomas in patients with persistent hyperparathyroidism, particularly in younger patients where such findings often signal MEN1. Surgeons must be knowledgeable about the anatomical variations of the parathyroid gland, genetic implications and to employ comprehensive imaging strategies to optimize surgical outcomes.

## Data Availability

The original contributions presented in the study are included in the article/**Supplementary Material**. Further inquiries can be directed to the corresponding author.
