# Supplementary figures and images for: Case Report: An undescended intravagal parathyroid adenoma: a rare cause of refractory primary hyperparathyroidism and severe osteoporosis
Source: Front Endocrinol (Lausanne). 2026 Feb 18;17:1783095. doi: 10.3389/fendo.2026.1783095 (PMC12956723; doi:10.3389/fendo.2026.1783095)

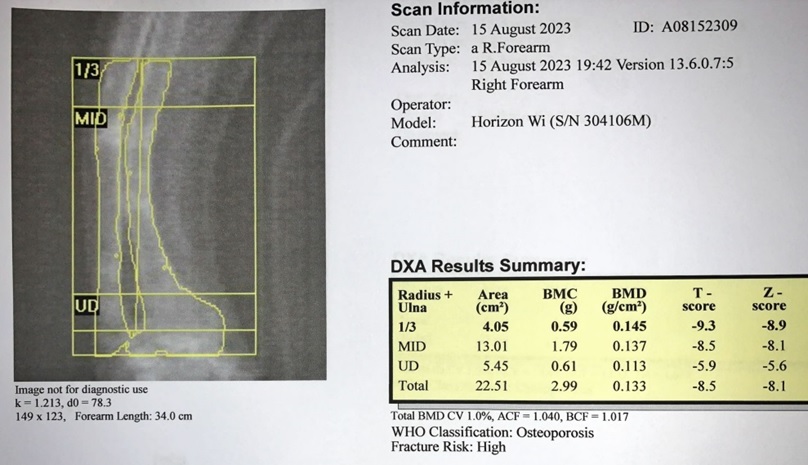

Supplement: Supplementary file 1 [file Image1.jpeg]

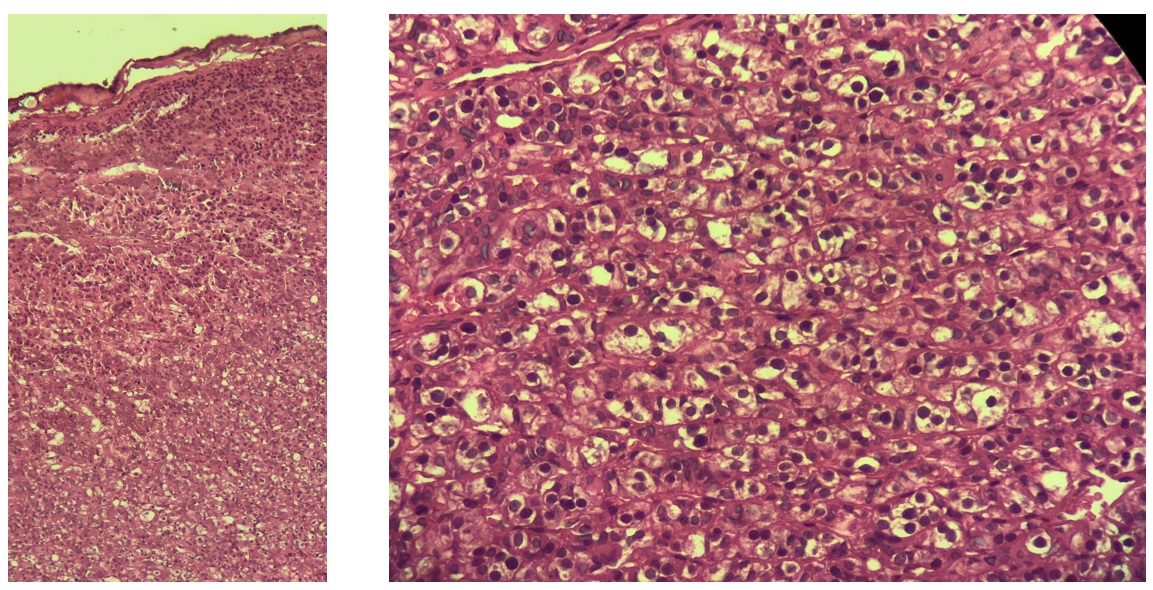

Supplement: Supplementary file 2 [file Image2.jpeg]

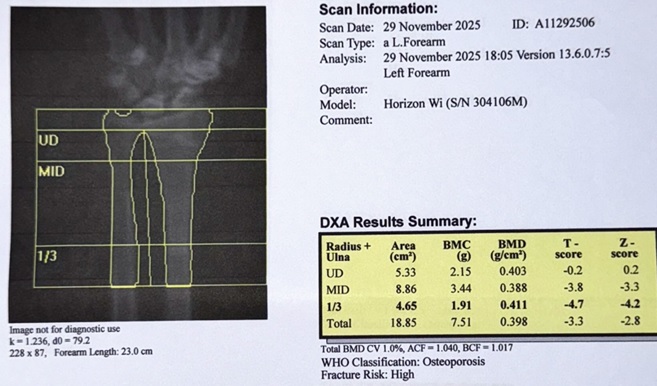

Supplement: Supplementary file 3 [file Image3.jpeg]
